# Untargeted Metabolomic Approach to Study the Impact of Aging on Salivary Metabolome in Women

**DOI:** 10.3390/metabo12100986

**Published:** 2022-10-18

**Authors:** Pauline Bosman, Valérie Pichon, Ana Carolina Acevedo, Laëtitia Le Pottier, Jacques Olivier Pers, Hélène Chardin, Audrey Combès

**Affiliations:** 1 Laboratoire des Sciences Analytiques, Bioanalytiques et Miniaturisation, UMR 8231 CBI CNRS, ESPCI Paris, PSL Université, 75005 Paris, France; 2 Sorbonne Université, 75006 Paris, France; 3 Laboratory of Oral Histopathology, Health Sciences Faculty, University of Brasilia, Brasília DF CEP 70910-900, Brazil; 4 Université Paris Cité, 75006 Paris, France; 5 LBAI, UMR 1227, Université de Brest, Inserm, 29200 Brest, France; 6 University Hospital of Brest, 29200 Brest, France

**Keywords:** LC-HRMS, salivary metabolome, aging

## Abstract

Despite the growing interest in salivary metabolomics, few studies have investigated the impact of aging on the salivary metabolome. The alterations in metabolic pathways that occur with aging are likely to be observed in pathologies affecting older people and may interfere with the search for salivary biomarkers. It is therefore important to investigate the age-related changes occurring in the salivary metabolome. Using reversed phase liquid chromatography and hydrophilic interaction chromatography coupled to mass spectrometry used in positive and negative ionization modes, the salivary metabolic profiles of young (22 to 45 years old) and older people (55 to 92 years old) were obtained. Those profiles were compared with the use of XCMS online to highlight the under or overexpression of some metabolites with aging. A total of 60 metabolites showed differential expression with age. The identification of 26 of them was proposed by the METLIN database and, among them, 17 were validated by standard injections. Aging seemed to affect most of the main metabolic pathways (amino acid metabolism, Krebs cycle, fatty acid synthesis, and nucleic acid synthesis). Moreover, most of the metabolites that were over- or under-expressed with age in this study have already been identified as being potential biomarkers of diseases affecting older people, such as in Alzheimer’s disease. Special attention should be paid in the search for biomarkers of pathologies affecting the elderly to differentiate age-related changes from disease-related changes.

## 1. Introduction

With increases in the aging of the population, the age structure of the world population has changed. Approximately 10% of the world’s population is now over the age of 60 and this figure will almost double by 2050 [1,2]. Aging leads to various changes in the human body that are more or less deleterious and can lead to loss of function and low resistance to chronic disease [2,3]. 

The oral fluid, more commonly known as saliva, is a complex biological fluid essential for maintaining oral health and nutrition [4]. Saliva is a viscous, slightly acidic liquid composed mainly of water and containing organic and inorganic compounds [5,6]. Saliva is secreted by the major (submandibular, sublingual, and parotid) and minor salivary glands [7]. With age, histological changes occur in the structure of the salivary glands, leading to a decrease in the proportion of acini and increase in the proportions of adipose and fibrous tissue. These histological changes naturally result in the hypofunction of the salivary glands, and subsequent decreases in secretion and alterations of the composition of saliva [1,8,9,10,11]. In addition, there is a decrease in the number of olfactory and gustatory receptors in the elderly, as well as a decrease in the neuronal stimulation of the glands, which leads to decreases in the intensity of stimuli and secretion reflexes, which then reinforce the hypofunction of the glands [10,11]. Some contradictions appear in the literature concerning the decrease in salivary flow. These contradictions are largely due to variations in study design. Indeed, some studies differentiate between groups of young and older people who are too close in age, other studies exclude all individuals taking medication and only consider healthy people, and others include minimal and hormone medications and/or people with disease. Finally, there are variations in the salivary collection methods, with studies based on unstimulated, stimulated, or parotid saliva. After a literature review, Affoo et al. [12] concluded that salivary flow rates in the elderly are significantly lower than in younger people, and this decrease is mainly attributed to the activity of the sublingual and submandibular glands. The parotid gland, on the other hand, seems to be less affected by age [1,8,9,10,11,12].

Despite the rise of metabolomics and growing interest in the salivary metabolome for biomarker discovery, only a few studies have been performed on the impact of aging on the salivary metabolome. Some authors have found alterations in several important metabolic pathways, such as glycolysis and gluconeogenesis, the pentose phosphate pathway, the tricarboxylic acid cycle, the urea cycle, nucleotide metabolism, glutathione metabolism, and acetylcholine metabolism [11,13]. Some alterations in amino acid metabolism may be associated with diseases that often develop with age, such as sarcopenia, which is a gradual loss of muscle mass and function, and osteoporosis, which is characterized by reduced bone strength [14,15]. Therefore, to discover biomarkers for pathologies affecting the elderly, we first need to characterize the natural aging process. 

Thus, the aim of the present work was to identify alterations in the salivary metabolome related to the natural aging process. As sex differences exist, especially in the aging process, we chose to focus our study on the salivary metabolome of women. Thus, the saliva of two groups of healthy women, young (22 to 45 years old) and older (55 to 92 years old), were analyzed. The metabolic profiles obtained by LC-HRMS analysis were then compared, to highlight the metabolites over- or under-expressed in saliva; these expression differences were correlated with age-related metabolic pathways and disease. 

## 2. Materials and Methods

### 2.1. Chemicals 

HPLC-grade ACN and MeOH were supplied by Carlo Erba (Val de Reuil, France) and formic acid by Sigma-Aldrich (Saint Quentin Fallavier, France). High-purity water was dispensed by a Milli-Q purification system (Millipore, Saint Quentin en Yvelines, France). Alanine, GABA, lysine, threitol, ornithine, tyrosine, phenylalanine, and fucose were purchased from Sigma Aldrich (St Louis, MO, USA). Pyroglutamic acid was purchased from EGA Chemie (Steinheim, Germany). Threonine, serine, glutamic acid, and leucine/isoleucine were purchased from Merck (Darmstadt, Germany). Succinic acid was purchased from Prolabo (Paris, France). Isovaleric acid was purchased from Fluka (St Louis, MO, USA). Hypoxanthine was purchased from Extrasynthese (Genay, France) and linoleic acid was purchased from Interchim (Montluçon, France).

### 2.2. Selection Criteria for Participants

To participate in the study, participants self-reported their age and whether or not they had any chronic pathologies or take any medications. This study involved a total of 50 healthy women who were divided into two groups: the control group, which included 24 women aged 22–45 years (32.8 ± 7.4), and the experimental group, including 26 women aged 55–92 years (61.5 ± 7.8). This study design would allow us to see the potential effects of aging on salivary metabolites. As aging is a phenomenon that can vary according to each individual and as menopause, which may be a major determinant of metabolomic alterations, happens between 45 and 55 years of age, a gap of 10 years was deliberately left between the two groups.

### 2.3. Collection and Storage of Salivary Samples

Our group previously reported on the importance of standardizing sample collection and treatment to allow for proper comparison of metabolomic profiles [16]. In view of the different partners, the samples were collected either by aspiration (24 samples) or by spitting (26 samples), two methods that were previously shown to give similar results for metabolomic analysis [16]. However, the following rules were defined to maximize standardization of sample collection: (i) All the volunteers were asked to refrain from having any food or drink or brushing their teeth for at least 2 h before saliva collection; (ii) The samples were collected without prior rinsing of the mouth; (iii) The collection time was set at 5 min; and (iv) Subjects were asked not to swallow, so that all oral fluid produced during the collection time would be collected. After collection, all saliva samples were centrifuged at 4 °C, 4500× *g* for 15 min and were stored at −20 °C until further analyses. Prior to LC-MS analyses, the samples were diluted by a factor 2 with ACN for HILIC and with a mixture of ACN/deionized water, 10/90, *v*/*v* for RP-LC. 

### 2.4. Instrumentation and Analytical Conditions 

Chromatographic separation and detection by mass spectrometry were performed according to Bosman et al. [16]. The analyses were performed using a liquid chromatograph (1260 Infinity, Agilent Technologies, Waldbronn Germany), coupled to a tandem mass spectrometer (microOTOF2, Bruker Daltonics, Bremen, Germany), and equipped with an electrospray ionization source. All data acquisitions were controlled using TOF Control software (version 3.4, Bruker Daltonics), and Hystar software (version 3.2, Bruker Daltonics) was used to interface the HPLC and MS systems. An Acclaim 120 C18 column (Thermo Scientific, Saint Quentin Fallavier, France, 150 × 2.1 mm, 3 µm) and an Acclaim HILIC 10 (Thermo Scientific, 150 × 2.1 mm, 3 µm) were used for RP-LC and HILIC separations, respectively. The temperature of columns was set at 40 °C, the mobile phase flow rate at 0.2 mL/min, and injection volume at 10 µL, for both chromatographic modes. A mobile phase composed of deionized water (A) and ACN (B) were both acidified with 0.1% of formic acid for RP-LC and HILIC methods, respectively. For RP-LC, a gradient consisting of 95% of A for 5 min, followed by an increase of 2%/min of B for 45 min, a plateau at 95% of B for 2 min, return to starting conditions, i.e., 5% of B, in 1 min, and ending with 10 min of equilibration was used. For HILIC, we used a gradient consisting of 98% of B for 5 min, followed by an increase of 0.3%/min of A for 45 min, then a plateau at 15% of A for 2 min, return to initial conditions in 1 min, and finally ending with 10 min equilibration. The MS detection was made alternatively in positive and negative mode in scan mode (*m*/*z* ranging from 50 to 500). The capillary voltage was set at 4000 V, nebulizer pressure at 20 psi, drying gas at 8 L/min, dry temperature at 200 °C, ion energy at 2.5 eV, and collision cell energy at 5 eV. A stepping in the basic mode that alternates every 0.25 s between 70 and 500 Vpp for RF collision and between 50 and 120 µs for the transfer time was used to facilitate the transfer of ions on the range of *m*/*z*. As previously demonstrated, the widest possible coverage of salivary metabolites, and thus, the most comprehensive comparison possible for metabolomic profiles was obtained when each saliva sample was analyzed using four analytical methods [16]. Two chromatographic separation modes (RP-LC and HILIC) coupled with detection by HRMS used in positive or negative ionization modes, were respectively named RP-LC+, RP-LC−, HILIC+, and HILIC−, having been thus implemented. The online software XCMS was then used to perform four comparisons, one for each analytical method used, between LC-MS profiles obtained in an untargeted way for women under 45 years and those obtained for women older than 55 years.

### 2.5. Quality Control for Untargeted Metabolomics

Each saliva sample was injected twice into the LC-MS system. After verifying that the two LC-MS injections of the same sample gave the same LC-MS profile, only one of the replicates was used to compare the LC-MS profiles between the two groups, using XCMS online [17]. Otherwise, a blank was inserted between each injection of a saliva sample to verify the absence of carry-over between the different samples. Finally, for quality control, we used a solution containing 90 compounds that had already been described in the literature and was confirmed by our group as being present in saliva [16], i.e., amino acids, fatty acids, and other small molecules, with *m*/*z* between 60.021 and 430.381 and belonging to a wide range of polarity (lop P values in the range −4.70–10.51). Indeed, in a previous study [16], we demonstrated that these compounds, which are representative of salivary metabolites in terms of molecular mass, structural family, and polarity, induced a response from the analytical system that was proportional to the analyte concentration and allowed us to obtain limits of quantification to the order of a hundred ng/mL. Thus, a solution containing the 90 selected standards was injected at the beginning, middle, and end of each sequence of injections in the LC-MS system, regardless of the method used (RP-LC or HILIC with MS detection in positive or negative mode). This allowed us to verify the absence of significant fouling (less than 25% decrease in the observed signal) of the MS detector. This control ensured the absence of bias in the measured signal, and thus, a proper comparison of the observed metabolomic profiles.

### 2.6. Data Analysis

The LC-HRMS profiles were converted into mzML files using Hystar software, version 3.2 (Bruker Technology, Bremen, Germany). They were then uploaded to XCMS Online (xcmsonline.scripps.edu) [17]. All LC-MS profiles were processed to extract the molecular features, using an allowance of 10 ppm on the experimental *m*/*z*, a minimum S/N ratio of 10, and the “center-wave” algorithm. The obi warp algorithm was used for the retention time correction and alignment, with a maximum of 5 s allowed for deviation and a step size of 0.5 *m*/*z*. 

### 2.7. Metabolic Pathways

The metabolites identified using the METLIN database [18] were searched for in the Human Metabolome Database (hmdb.ca), KEGG network (genome.jp), and PubChem (pubchem.ncbi.nlm.nih.gov). Stock solutions with concentrations ranging from 0.1 to 1 mg/mL were prepared in appropriate solvents (water, ACN, MeOH, and mixtures of these solvents), depending on the solubility of the compound, and stored at 4 °C. Working solutions were prepared from these stock solutions at concentrations between 0.25 and 18 µg/mL and injected. 

## 3. Results and Discussion

### 3.1. Comparison of the LC-MS Profiles of Young and Elderly Women

**Figure 1:**
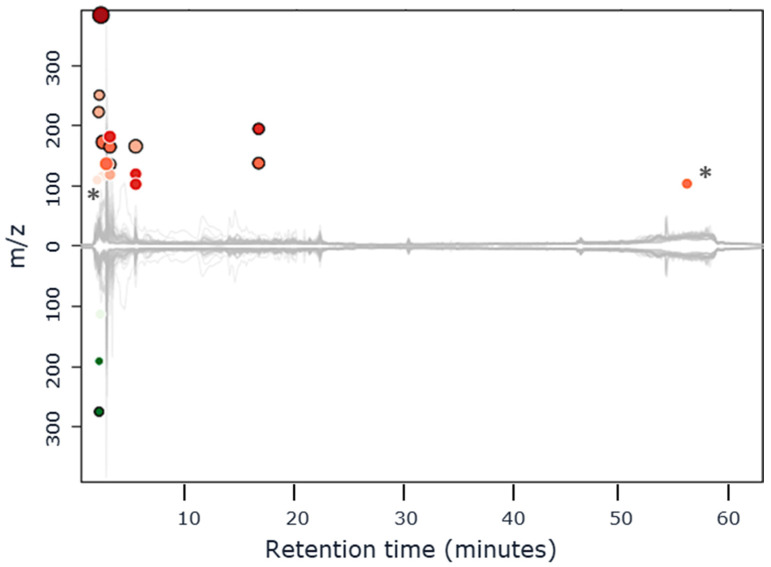
Cloud plots showing under- and over-expressed features with age in RPLC/MS+. The red bubbles over the chromatogram represent the over-expressed features and the green bubbles under the under-expressed. The bigger the bubble is, the bigger the fold change, and the darker the bubble, the more significative the *p*-value. * corresponds to features that were removed after manual verification.

Due to the alignment algorithm, XCMS detected hundreds of features (defined by the same retention time during chromatographic separations and the same *m*/*z* ratio during the MS analysis) that were present in both groups. Among these features, a significant inter-group difference (up- or down-regulated, fold change minimum set at 1.5, and *p*-value inferior to 0.05) was observed using MS detection in positive mode for 26 features after RP-LC separation, as shown in Figure 1 in a cloud plot representing under- and over-expressed compounds in RP-LC-MS+ (results not shown for the three other methods), and 13 features after HILIC separation. Then, the extracted ion chromatograms (EIC) for the 39 corresponding features were generated and manually verified. Two features were just noise and did not correspond to a chromatographic peak, so they were eliminated. An additional step of manual verification of the raw mass spectra, corresponding with a given retention time, made it possible to eliminate six additional features which were eluted from the chromatographic column with the same retention time and that corresponded with an ion in mass spectra belonging to the isotopic pattern of another ion already listed under its monoisotopic form. These verifications were necessary and supported that software should not be blindly used in without manual verification. The total list of 31 features (couple of *m*/*z* and retention time), showing statistical differential expression with age (12 were highlighted from the comparison of the LC-MS profile obtained after HILIC versus 19 highlighted after RP-LC separation and detection in positive mode) is given in Table S1 (Supplementary material). The same approach was applied to the LC-MS profile obtained after separations on RP-LC and HILIC mode coupled with a MS detection in negative mode. XCMS online highlighted 24 and 15 features after RP-LC and HILIC separation, respectively. The manual verifications of EIC and raw mass spectra led us to eliminate four features that were noise and did not correspond to a chromatographic peak from HILIC analysis and three that did not correspond to monoisotopic mass from RP-LC−. The list of the 32 features (couple of *m*/*z* and retention time), showing significative differential expression with age (11 after HILIC and 21 after RP-LC separation), is shown in Table S1 (Supplementary material).

**Figure 2:**
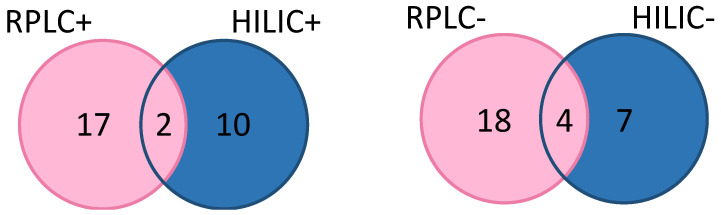
Venn diagram showing the number of features detected according to the analytical method used. RPLC+: reversed-phase liquid chromatography coupled to mass spectrometry used in positive ionization mode; RPLC−: reversed-phase liquid chromatography coupled to mass spectrometry used in negative ionization mode; HILIC+: hydrophilic interaction liquid chromatography coupled to mass spectrometry used in positive ionization mode; HILIC−: hydrophilic interaction liquid chromatography coupled to mass spectrometry used in negative ionization mode.

Among these 63 features, only seven ions (same *m*/*z*) were found to be in common with the RP and HILIC separations (two and four in positive and negative modes, respectively, as seen in Figure 2, and one in common between RP-LC+ and HILIC−). This proves the complementarity of the four analytical methods and the usefulness in implementing them all. A closer look at these ions (they are highlighted in Table S1) demonstrated that their differences in expression were similar (same direction of variation and same magnitude of change), regardless of the chromatographic separation mode used. For example, the ion with a *m*/*z* of 274.875 detected in the MS positive ionization mode decreased with age by a factor of 1.7 and 1.5 depending on the chromatographic separation in HILIC and RP-LC, respectively.

Furthermore, one of the known effects of aging is decreasing the salivary flow rate, and thus, the volume of saliva produced during collection time [10,11], which could lead to a bias in the comparison of metabolic profiles. This could lead to = generalized over- or under-expression of all the compounds detected in the saliva of elderly women. However, it is interesting to note that, among the 57 features with different expression levels highlighted listed in Table S1 (Supplementary material), 35 are overexpressed and 22 are under-expressed in the group of elderly people. This clearly demonstrates the absence of such bias in this study.

### 3.2. Identification of the over = or Under-Expressed Compounds

**Figure 3:**
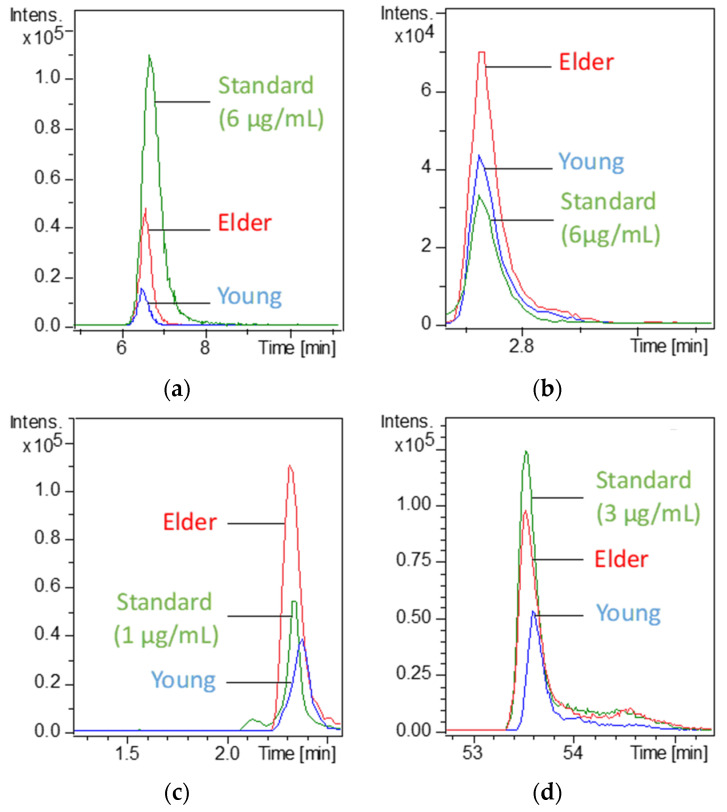
Extracted ion chromatograms for succinic acid (*m*/*z* 117.020), hypoxanthine (*m*/*z* 137.047), and linoleic acid (*m*/*z* 279.240) analytical standards and for the corresponding compounds in the saliva of young and elderly people: (**a**) succinic acid in HILIC/MS−, (**b**) succinic acid in RPLC/MS+, (**c**) hypoxanthine in RPLC/MS+, and (**d**) linoleic acid in RPLC/MS−.

To identify these 57 features showing differential expression with age, the METLIN database was used and identification was proposed for 26 features. For 17 of them, analytical standards were available, and they were thus injected using the same analytical method(s) that revealed their difference in expression with aging. The superposition of the EIC corresponding to the analytical standard with that of the corresponding compound (same *m*/*z*) in the saliva of young and elderly people confirmed their identification (same *m*/*z* and same retention time). Figure 3 shows an example of these EIC overlays for three compounds: succinic acid (*m*/*z* 117.090 or 119.051 according to the detection mode), hypoxanthine (*m*/*z* 137.047), and linoleic acid (*m*/*z* 279.240). 

The 17 compounds for which identification could be confirmed using these comparisons with available analytical standards are summarized in Table 1. The nine remaining compounds are listed in Table 2 with their respective putative identification.

### 3.3. Evolution of Saliva Metabolome with Age and Potential Affection of Metabolism

The Human Metabolome DataBase (HMDB) verified that all the compounds listed in Table 1 had already been described as present in saliva. Among the 17 compounds overexpressed in the saliva of the elderly women, more than half were amino acids or amino acid derivatives. Indeed, nine of them were free proteinogenic amino acids (alanine, serine, threonine, leucine/isoleucine, lysine, glutamic acid, phenylalanine, and tyrosine). Among these amino acids, four were amino acids (leucine/isoleucine, lysine, phenylalanine, and threonine) that could not be produced by the human organism (essential amino acids) and therefore need to be provided by the diet. Their presence in higher concentrations in the saliva of elderly women was a sign of an imbalance between protein synthesis (anabolism) and proteolysis (catabolism), in favor of proteolysis. This imbalance has been described as a characteristic of sarcopenia, which affects many elderly people [15,19]. The higher concentration of amino acids in the saliva of elderly women differs from the results obtained in Teruya et al. [13] study, in which the levels of amino acids decreased with age, but correlates partially with the results from Tanaka et al. [20], who observed higher levels of lysine. Ornithine is a non-proteinogenic amino acid that plays a crucial role in the urea cycle. This allows the excretion of the nitrogen generated by the breakdown of amino acids in protein and other nitrogen-containing molecules from the body in urine. It is also interesting to note the overexpression of pyroglutamic acid, an uncommon amino acid derivative in which the free amino group of glutamic acid, which is itself overexpressed in the saliva of elderly people, cyclizes to form a lactam. It has been shown in an animal model that pyroglutamic acid led to the release of GABA from the cerebral cortex [21], a neurotransmitter also overexpressed in the saliva of elderly women.

Hypoxanthine is a naturally occurring purine derivative (by spontaneous deamination of adenine) and an intermediate in the metabolism of adenosine and in the formation of nucleic acids by the nucleotide salvage pathway. 

Concerning the two fatty acids belonging to the fatty acyl family that were overexpressed in saliva, linoleic acid is a doubly unsaturated fatty acid, also known as an omega-6 fatty acid. It is an essential fatty acid that is used in the biosynthesis of prostaglandins (via arachidonic acid) and cell membranes [22]. Isovaleric acid is a natural methyl-branched fatty acid found in a wide variety of plants and essential oils.

Fucose is the only carbohydrate that was detected as being overrepresented in the saliva of elderly women. It is a monosaccharide, hexose deoxy sugar, and it is a common component of many N- and O-linked glycans and glycolipids. As a free sugar, L-fucose is normally found at very low levels of concentration in the saliva of mammals [23]. However, high concentrations of free L-fucose in serum and urine have been described to be a biomarker for different pathologies that affect the liver (cancer, cirrhosis, alcoholic liver disease, and gastric ulcers) [24,25]. The increase in this sugar in the saliva of elderly people could therefore be linked to a decrease in the metabolic activity of the liver in connection with aging [26]. In the same pathway, threitol is the main end-product of D-xylose metabolism in humans.

Another major metabolic pathway that seems to be affected by aging is the Krebs cycle. Succinic acid, a dicarboxylic acid that is an important component of the Krebs cycle, and thus, of the formation of adenosine triphosphate (ATP), was found in higher concentrations in the saliva of elderly women. Succinic acid acts as a signaling molecule reflecting the cellular metabolic state and regulates many processes in living cells. It has also been shown that succinic acid is an oncometabolite [27] that promotes tumor development and progression by inhibiting the activity of several 2-oxoglutarate-dependent enzymes. This superfamily of enzymes participates in many biological processes, including adaptation to hypoxia, epigenetic reprogramming, or the maturation and stability of collagen fibers.

Given all the metabolites identified (putatively or confirmed), aging seems to affect most of the main metabolic pathways of the organism (proteolysis, Krebs cycle, fatty acid synthesis, and nucleic acid synthesis). However, almost all of the salivary metabolites that were found to be overexpressed with age have already been suggested to be associated with pathologies of the oral sphere (periodontitis, dental caries, oral cancers, etc.) or with systemic diseases, such as pancreatic, liver or breast cancers, Alzheimer’s disease, or Lewy body disease [26,28,29]. 

## 4. Conclusions

The metabolic profiles of the saliva of women younger than 45 years and older than 55 years were obtained with the use of RP-LC and HILIC, coupled to HRMS, used in positive and negative ionization modes. When these metabolic profiles were compared using the statistical tool XCMS Online, our results showed that 35 features (coupled with retention time and *m*/*z*) were overexpressed and 22 under-expressed in older women. Using the METLIN database, an identification was proposed for 26 metabolites and confirmed for 17 of them. Among these 17 metabolites that showed differential expression with aging, 11 amino acids and amino acid derivatives, a purine derivative, 2 carbohydrate and carbohydrate derivatives, 2 fatty acids, and a dicarboxylic acid were found. Given the identified metabolites, the metabolic pathways that seemed to be affected with age were mainly amino acid metabolism, the Krebs cycle, fatty acid metabolism, and nucleic acid synthesis. Some of the metabolites identified in this study as being over- or under-expressed with age have been put forward as potential biomarkers of pathologies of the elderly, which raises questions about whether these biomarkers are related to age or to the pathology. Our results show, therefore, the essential need to characterize the natural process of aging before the identification of biomarkers for pathologies affecting older people.

## Figures and Tables

**Table 1:** Salivary metabolites for which the identification was confirmed.

Measured *m*/*z*	Fold Change (over Expressed)	Raw Formula	Confirmed Identification *	Log P	Analytical Method	Retention Time (min)
88.041	1.8	C_3_H_7_NO_2_	Alanine	−3.2	RPLC−	1.73
102.058	2.7	C_4_H_9_NO_2_	γ-aminobutyric acid (GABA)	−2.3	RPLC−	1.75
103.056	2.4	C_5_H_10_O_2_	Isovaleric acid	1.21	RPLC+	5.04
104.037	2.6	C_3_H_7_NO_3_	Serine	−3.9	RPLC−	1.69
119.051	1.9	C_4_H_6_O_4_	Succinic acid **	−0.4	RPLC+ HILIC−	2.66 6.61
117.020	3.0					
120.083	2.3	C_4_H_9_NO_3_	Threonine	−3.5	RPLC+	5.04
123.046	2.0	C_4_H_10_O_4_	Threitol	−2.5	RPLC+	2.65
128.037	4.6	C_5_H_7_NO_3_	Pyroglutamic acid	−0.89	RPLC−	2.54
130.089	3.1	C_6_H_13_NO_2_	Leucine/Isoleucine	−1.6	RPLC−	3.12
131.083	2.3	C_5_H_12_N_2_O_2_	Ornithine	−3.7	RPLC−	1.68
137.047	3.0	C_5_H_4_N_4_O	Hypoxanthine	−0.05	RPLC+	2.32
145.101	2.8	C_6_H_14_N_2_O_2_	Lysine	−3.2	RPLC−	1.56
146.050	2.5	C_5_H_9_NO_4_	Glutamic acid	−3.2	RPLC−	1.75
165.056	2.2	C_6_H_12_O_5_	Fucose	−1.9	RPLC+	2.65
166.087	2.5	C_9_H_11_NO_2_	Phenylalanine	−1.2	RPLC+	5.03
182.083	2.6	C_9_H_11_NO_3_	Tyrosine	−1.5	RPLC+	2.65
279.236	1.9	C_18_H_32_O_2_	Linoleic acid	6.42	RPLC−	53.75

* The identification of compounds was confirmed by comparing the *m*/*z* and retention time of the unknown compound with those of an analytical standard injected under the same analytical conditions (LC separation and MS detection mode). ** For these compounds, the identification was confirmed with two different analytical methods.

**Table 2:** Potential salivary metabolites identified with METLIN.

Measured *m*/*z*	Fold Change (over or under Expressed)	Raw Formula	Putative Identification (Error in ppm)	Analytical Method	Retention Time (min)
136.076	2.1 (Over)	C_8_H_9_NO	Phenylacetamide (5)	RPLC+	2.65
138.068	2.0 (Over)	C_6_H_13_N	Cyclohexylammonium (4)		16.41
173.094	2.7 (Over)	C_10_H_14_O	Thymol (4)		2.03
195.088	1.9 (Over)	C_8_H_10_N_4_O_2_	Caffein (4)		16.41
223.026	1.9 (Over)	C_11_H_8_N_2_O	Fuberidazole (3)		1.62
384.150	4.1 (Over)	C_14_H_25_NO_11_	N-acetyllactosamine or Β-mannose-N-acetylglucosamine (1)		1.82
98.984	2.5 (Over)	H_3_PO_4_	Phosphoric acid (4)	HILIC+	2.27
256.103	2.6 (Over)	C_9_H_15_N_5_O_4_	Tetrahydroneoptin (10)	RPLC−	2.27

## Data Availability

All data and materials are stored in the Laboratory of Bioanalytical Sciences and Miniaturization (LSABM), UMR CBI 8231, ESPCI Paris, CNRS, PSL University, Paris, France. The data are not publicly available due to ethical concerns.

## Supplementary Materials

The following supporting information can be downloaded at: https://www.mdpi.com/article/10.3390/metabo12100986/s1, Table S1: *m*/*z* values of features statistically over- or under-expressed with age after analysis in RP-LC or HILIC coupled to HRMS in positive or negative mode.
