# Supplementary material for: Untargeted Metabolomic Approach to Study the Impact of Aging on Salivary Metabolome in Women
Source: Metabolites. 2022 Oct 18;12(10):986. doi: 10.3390/metabo12100986 (PMC9612358; doi:10.3390/metabo12100986)
Supplement: Supplementary file 1 [file metabolites-12-00986-s001.zip › metabolites-1959737-supplementary.pdf]

SM1: ***m/z* values of features statistically over- or under-expressed with age after analysis in RP-LC or HILIC coupled to HRMS in positive or negative mode**

m/z observed	Fold change*	OVER or UNDER expressed	p-value	Method	Retention time (min)
102.058	2.7	OVER	0.009	RP-LC-	1.75
103.056	2.4	OVER	0.018	RP-LC +	5.04
104.037	2.6	OVER	0.001	RP-LC-	1.69
106.95	2.5	UNDER	0.002	HILIC +	2.46
112.9	1.8	UNDER	0.05	RP-LC +	1.76
112.985**	1.5	UNDER	0.0001	RP-LC-	1.59
112.986**	1.3	UNDER	0.044	HILIC-	2.32
116.071**	2.4	OVER	0.049	HILIC +	38.5
116.073**	1.9	OVER	0.043	RP-LC +	1.9
117.02**	3	OVER	0.01	HILIC-	6.61
119.051**	2.1	OVER	0.039	RP-LC +	2.66
120.083	2.3	OVER	0.02	RP-LC +	5.04
123.046	2	OVER	0.046	RP-LC +	2.65
128.037	4.6	OVER	0.051	RP-LC-	2.54
130.089	3.1	OVER	0.016	RP-LC-	3.12
131.083	2.3	OVER	0.016	RP-LC-	1.68
132.032	1.9	OVER	0.02	RP-LC-	1.74
136.076	2.1	OVER	0.039	RP-LC +	2.65
137.047	3	OVER	0.029	RP-LC +	2.32
138.068	2	OVER	0.022	RP-LC +	16.41
145.101	2.8	OVER	0.003	RP-LC-	1.56
146.05	2.5	OVER	0.01	RP-LC-	1.75
165.056	2.2	OVER	0.025	RP-LC +	2.65
166.087	2.5	OVER	0.033	RP-LC +	5.03
173.094	2.7	OVER	0.03	RP-LC +	2.03
182.083	2.6	OVER	0.012	RP-LC +	2.65
190.913	1.5	UNDER	0.001	RP-LC +	1.65
195.088	1.9	OVER	0.014	RP-LC +	16.41
223.026	1.9	OVER	0.037	RP-LC +	1.62
251.032	1.7	OVER	0.031	RP-LC +	1.67
256.103	2.6	OVER	0.015	RP-LC-	2.27
258.898	2.3	UNDER	0.002	HILIC +	2.46
264.844	2.5	OVER	0.029	HILIC +	2.47
264.933**	1.8	UNDER	7.0E-05	RP-LC-	1.57
264.939**	1.7	UNDER	0.017	HILIC-	2.32
274.872**	1.7	UNDER	0.041	HILIC +	2.44
274.875**	1.5	UNDER	0.0001	RP-LC +	1.65
279.236	1.9	OVER	0.019	RP-LC-	53.75
293.146	3.8	OVER	0.038	RP-LC-	16.03
316.946	6	UNDER	0.043	RP-LC-	1.57
332.920**	2.8	UNDER	0.001	RP-LC-	1.57

332.928**	2.1	UNDER	0.006	HILIC-	2.32
342.859	2.2	UNDER	0.006	HILIC +	2.44
348.895**	1.6	UNDER	0.001	RP-LC-	1.57
348.903**	1.7	UNDER	0.024	HILIC-	2.32
357.039	1.9	UNDER	0.003	HILIC-	35.87
372.819	2.7	OVER	0.029	HILIC-	2.32
373.013	1.6	UNDER	0.029	HILIC-	35.81
380.857	2.8	OVER	0.024	RP-LC-	1.58
382.032	2	UNDER	0.036	RP-LC-	1.85
384.15	4.1	OVER	0.004	RP-LC +	1.82
394.872	2.9	UNDER	0.011	HILIC +	2.46
400.907	3.6	UNDER	0.004	RP-LC-	1.56
410.846	2.1	UNDER	0.006	HILIC +	2.45
416.897	2.2	UNDER	0.005	HILIC-	2.32
422.836	1.7	OVER	0.038	HILIC-	2.34
462.858	4.5	UNDER	0.005	HILIC +	2.46
478.833	2.4	UNDER	0.003	HILIC +	2.46
494.807	2.1	UNDER	0.016	HILIC +	2.44
73.028	3	OVER	0.01	HILIC-	6.61
88.041	1.8	OVER	0.044	RP-LC-	1.73
92.926	3.2	UNDER	0.026	RP-LC-	1.72
98.984	2.5	OVER	0.014	HILIC +	2.27
102.058	2.7	OVER	0.009	RP-LC-	1.75

* Increasing or decreasing fold change between the group of elderly people and the control group

** Features that presents differential expression with age and that were found both in RPLC and HILIC
